# Comparative study of upper lip frenectomy with the CO2 laser versus the Er, Cr: YSGG laser

**DOI:** 10.4317/medoral.17373

**Published:** 2011-12-06

**Authors:** Jordi Pié-Sánchez, Antonio J. España-Tost, Josep Arnabat-Domínguez, Cosme Gay-Escoda

**Affiliations:** 1 DDS. Resident of the Master of Oral Surgery and Implantology. Barcelona University Dental School; 2 DDS. MD, PhD. Associate Professor of Oral Surgery. Professor of the Master of Oral Surgery and Implantology. Director of the Master in Lasers in Dentistry. Coordinator of the European Master’s Degree in Oral Laser Applications (EMDOLA). Barcelona University Dental School. Investigator of the IDIBELL Institute; 3 DDS. MD, PhD. Associate Professor of Oral Surgery. Co-director of the Master in Lasers in Dentistry. Barcelona University Dental School. Investigator of the IDIBELL Institute; 4 MD, DDS, PhD. Chairman and Full Professor of Oral and Maxillofacial Surgery. Director of the Master of Oral Surgery and Implantology. Barcelona University Dental School. Coordinating Investigator of the IDIBELL Institute. Head of the Department of Oral and Maxillofacial Surgery, Teknon Medical Center. Barcelona, Spain

## Abstract

Objectives: To compare upper lip frenulum reinsertion, bleeding, surgical time and surgical wound healing in frenectomies performed with the CO2 laser versus the Er, Cr:YSGG laser. Study design: A prospective study was carried out on 50 randomized pediatric patients who underwent rhomboidal resection of the upper lip frenulum with either the CO2 laser or the Er,Cr:YSGG laser. Twenty-five patients were assigned to each laser system. All patients were examined at 7, 14, 21 days and 4 months after the operation in order to assess the surgical wound healing. Results: Insertion of the frenulum, which was preoperatively located between the upper central incisors, migrated to the mucogingival junction as a result of using both laser systems in all patients. Only two patients required a single dose of 650 mg of paracetamol, one of either study group. CO2 laser registered improved intraoperative bleeding control results and shorter surgical times. On the other hand, the Er,Cr:YSGG laser achieved faster healing. Conclusions: Upper lip laser frenectomy is a simple technique that results in minimum or no postoperative swelling or pain, and which involves upper lip frenulum reinsertion at the mucogingival junction. The CO2 laser offers a bloodless field and shorter surgical times compared with the Er,Cr:YSGG laser. On the other hand, the Er,Cr:YSGG laser achieved faster wound healing.

** Key words:**Frenectomy, upper lip frenulum, CO2 laser, Er,Cr:YSGG laser, laser.

## Introduction

Oral frenula are bandlike formations of congenital origin located on the midline, which are composed of fibrous, muscular or fibromuscular tissue, and are covered with a mucosal membrane. They are regarded as anatomical formations that, under normal conditions, do not have pathological consequences. However, in some cases they can present clinical problems, fundamentally of orthodontic, prosthetic, phonetic or periodontal nature.

The upper lip frenulum is an oral mucosal membrane extending from the internal surface of the upper lip to its insertion on the midline of the attached interincisal gingival tissue of the upper maxilla. In some cases, the frenulum descends to the alveolar margin and inserts at the interdental papilla in the palatal vault. Two situations can be diferentiated from the clinical perspective. In children, these frenula mainly cause interincisal diastemas, which in turn require orthodontic treatment. In adult patients, frenula give rise to prosthetic problems, since they interfere with the retention and/or stability of the denture, and cause irritation.

**Figure 1:**
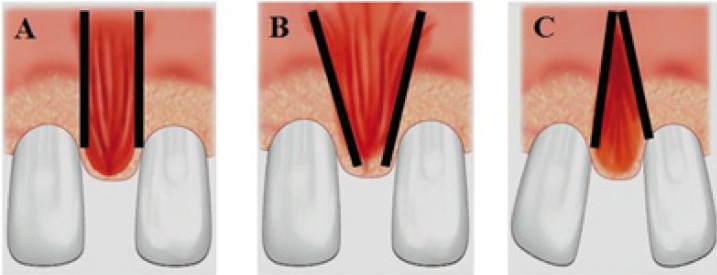
Classification of upper lip frenula according to Monti. A) Elongated frenulum with parallel margins. B) Triangular frenulum with apical base. C) Triangular frenulum with coronal base.

Different classifications of lip frenula have been described on the basis of their morphological characteristics. Monti distinguishes three types of frenula (Fig. 1):

• Elongated presentations with parallel left and right margins.

• Triangular frenula in which the base coincides with the vestibular sulcus.

• Triangular frenula with their base at the lowest position.

As to those presentations characterized by an important associated muscular component, the upper lip frenula can cause certain anomalies or problems, such as interincisal diastema, denture-related problems, periodontal disease secondary to retained or impacted food, oral hygiene difficulties, and impairment of lip mobility and/or a short lip (with involvement of lip sealing) (1,2).

The diagnosis is based on the aforementioned clinical data, with attention focused mainly on the presence of an interincisal diastema and a positive papilla sign. A radiological study is also required to discard other possible causes of diastemas, such as mesiodens, odontoma or root cysts, among other causes, and assess the characteristics of the interincisal bone. A panoramic X-ray study is recommended in this context, together with both a periapical X-ray of the interincisal zone and a vertex occlusal X-ray exploration to evaluate the relationship with the nasopalatine duct (1).

Many authors (3-6) have described the use of different lasers in oral soft tissue surgery. The advantages of the surgical laser treatment versus the cold scalpel are as follows: a bloodless surgical field (using the CO2 laser), no need for suturing because healing is by second intention, and postoperative pain and swelling are less intense or even absent.

In the last few years, the CO2 laser has been successfully used in oral soft tissue surgery procedures, such as gingival resections, gingivoplasties, soft tissue biopsy samples, frenectomies, treatment of lymphangiomas, and crown lengthenings (7-9). Because of their special characteristics, the CO2, the Er:YAG and the Er,Cr:YSGG lasers have been the systems most widely used to perform frenectomies.

The main objective of the present study was to compare the upper lip frenulum reinsertion following frenectomies performed with the CO2 laser or with the Er,Cr:YSGG laser, especially because of the absence of publications on this subject and in the interest of the predictability of results obtained when using those techniques. We also compared bleeding and treatment time, wound healing and postoperative pain and swelling in frenectomies performed with both types of lasers.

## Material and Methods

The study involved 50 pediatric patients scheduled for simple (rhomboidal) removal of the upper lip frenulum. The patients were referred to our Service of Oral Surgery from different departments of the University of Barcelona Dental Clinic (Spain). Patients requiring upper lip lengthening or bone surgery were excluded from the study. A case history was compiled, and panoramic X-rays and a vertex occlusal X-ray study were requested.

Distances between the insertion of the frenulum and the mucogingival junction (MGJ), as well as between the vertex of the interincisal papilla and the MGJ were measured before surgery in all cases with a caliper.

All patients were randomized to surgery with either the CO2 laser or the Er,Cr:YSGG laser (each group containing 25 patients).

The amount of anesthetic used in each case was 0.6 ml (1/3 of the carpule contents) of articaine 4% with epinephrine 1:200,000. The palatine interincisal papilla and the surgical area were anesthetized using the bilateral vestibular infiltration technique; the anesthetic needle was subsequently used to check the presence, or absence, and thickness of soft tissue at the level of the inter-maxillary suture.

The duration of the surgery procedure and intraoperative bleeding events (bloodless, slight bleeding, normal bleeding, and severe bleeding) was recorded in all cases.

The CO2 laser used in the study was the Sharplan® 1020 (Sharplan, Tel-Aviv, Israel), 10600 nm wavelength used in the focused continuous wave mode, a power rating of 5 W, a spot diameter of 0.8 mm, and a power density of 1000 W/cm2. The Er,Cr:YSGG laser used was the Waterlase® (Biolase Technology, Irvine, CA, USA), 2780 nm wavelength, a pulse duration between 140 and 200 µs, and a 20 Hz frequency. Power settings used were 1.5 W, 12% water and 8% air, and a spot of 0.6 mm in diameter by means of a G-4 tip (600 µm) and an straight handpiece with an energy density per pulse of 26.54 J/cm2.

The same surgical technique was used with both lasers: follow the vertical axis of the frenulum until the wound presented a linear shape. At this point the laser was applied transversely until the wound took a rhomboidal shape. Then, the palatine limit of the papilla was treated in those cases in wich the frenulum showed a low insertion.

Patients were instructed to keep a complete oral hygiene throughout the postoperative period. Antibiotics were not prescribed in any case, and patients were advised against mouth-rinses. Paracetamol 650 mg was administered 8-hourly to patients who complained of pain during the post operative period.

Patients should be advised not to be alarmed by the aspect that the wound may develop in the event that a white exudate showed up during the second-intention-healing of the wound.

All patients were seen 7, 14, 21 days and 4 months after surgery to assess surgical wound healing. The distance between the MGJ to the frenulum insertion and the vertex of the interincisal papilla was measured at 4 months after treatment. Swelling was assessed by questioning children’s parents or guardians.

The statistical analysis of the results was done using the Statistical Package for Social Sciences version 12.0 for Microsoft Windows (SPSS v. 12.0; SPSS Inc., Chicago, USA), license granted to the Barcelona University.

## Results

Of the group of 50 patients, 44% were male children (n=22). Mean patient age was 11.3 years, with a standard deviation (SD) of 0.8 years. Patients were referred to our Service of Oral Surgery from other departments of the University of Barcelona Dental Clinic (Spain), with a greater number of subjects referred from the Orthodontics department.

All permanent upper incisors had fully erupted at the time of surgery. However, in 28% (n=14) of cases right and left canines had not yet erupted.

The application of traction applied on the upper lip revealed a positive papilla sign in 46% (n=23) of cases. No diastema was observed in 26% (n=13) of patients. Mean diastema size was 1.78 mm (SD 1.72). 38% (n=19) of frenula were inserted at the level of the interincisal papilla (mean 1.56 mm, SD 1.53 mm).

Some fibers were detected during surgery at the level of the intermaxillary suture level in 40% (n=20) of cases. The mean duration of surgery procedures with the CO2 laser was 49.50±13.02 seconds, versus 162.56±56.70 seconds with the Er,Cr:YSGG laser. The mean irradiation time with the CO2 laser was 30.44±8.88 seconds, versus 77.35±19.04 seconds with the Er,Cr:YSGG laser – this represents a mean total tissue irradiation of 152.20 J and 116.03 J, respectively. The hemostatic efficacy of the Er,Cr:YSGG laser was more limited (moderate intraoperative bleeding) than that achieved with the CO2 laser (bloodless surgery).

Only two patients required a single dose of 650 mg of paracetamol to treat a mild postoperative pain; one patient from either group. None of the cases showed swelling of tissues adjacent to the surgical area.

When comparing results from the CO2 laser group with those of the Er,Cr:YSGG laser group, follow-up controls revealed that wound epithelization was complete at days 21 and 14, respectively. So it established that healing is faster with the Er,Cr:YSGG laser.

**Figure 2:**
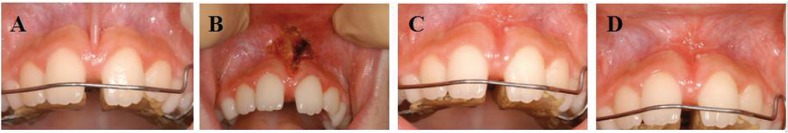
Upper lip frenectomy performed with the CO2 laser. A) Preoperative view. B) Immediate postoperative view. C) View 21 days after surgery. D) View 4 months after surgery.

**Figure 3:**
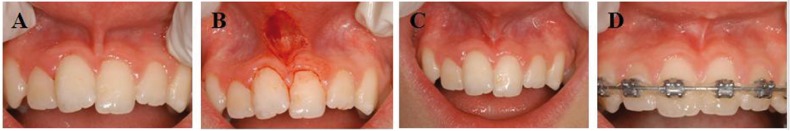
Upper lip frenectomy performed with the Er,Cr:YSGG laser. A) Preoperative view. B) Immediate postoperative view. C) View 21 days after surgery. D) View 4 months after surgery.

An evaluation made 4 months after surgery revealed that the insertion of frenula, which were preoperatively located between the upper central incisors level (mean 2.76 mm and 2.84 mm of distance with respect to the MGJ concerning both the CO2 and the Er,Cr:YSGG laser study groups, respectively), had migrated to the MGJ in all patients, regardless of the laser system used ( Table 1) (Figs. 2,3).

**Table 1:**
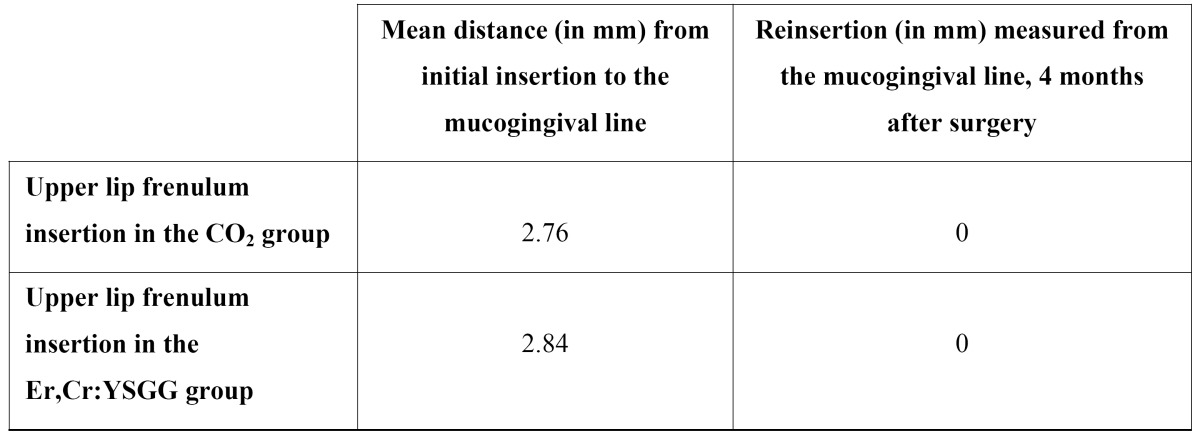
Student t-test comparison of the mean differences between the CO2 laser and the Er,Cr:YSGG laser in relation to upper lip frenulum insertion at 4 months.

## Discussion

Different cold-scalpel techniques were used to perform frenectomies before the introduction of laser techniques in oral surgery. These techniques were mainly simple or rhomboidal excisions, V-Y -plasties and Z -plasties (10,11).

Simple exeresis involves sectioning, or transverse sectioning, the frenulum, and subsequent suture. Simple exeresis, V-Y-plasty technique and apical positioning of the frenulum leave a scar in the same direction of the frenulum, whereas upper lip lengthening is not achieved; as a result, these techniques are contraindicated in those cases in which short lips exhibit an impaired lip seal.

Therefore, the technique of choice for upper lip frenulum surgery in short upper-lip patients is Z-plasty, since frenulum removal leaves a mucosal scar in another direction, and because the upper lip lengthening can be achieved by increasing the vestibular depth.

The introduction in recent years of surgical lasers has led to new alternative treatments. Compared with the conventional technique treatments, lasers offer new perspectives due to their differentiating characteristics (12).

In frenectomies, the main advantages of surgical lasers include the achievement of a precise and clean surgery, and shorter surgery times compared with conventional techniques (10). The CO2 laser vaporizes tissues and eliminates bleeding, since the procedure enables a good hemostasis of superficial vessels. Moreover, suturing is not required, since the wound is left open and heals by second intention due to the production of granulation tissue and re-epithelization, which occurs from the margins of the surgical wound to its center (5,13). However, it is not recomended to use laser techniques to perform frenectomies in patients with a short lip, or when deepening of the vestibular sulcus is needed. In such cases cold-scalpel Z-plasty should be preferred (11). The anesthetic technique is not different from that used to resect lip frenula with a cold scalpel, though the amount of anesthesia needed is smaller than with classical procedures.

Both, the diode (14) and the Nd:YAG lasers (4,15,16), are also useful for oral soft tissue surgery, frenectomies included, but they produce a greater thermal effect on adjacent tissues.

In our study, the surgical procedure was well tolerated by all the patients, and there were no adverse effects or complications either during surgery or after it.

Care is required to avoid damaging nasopalatine neurovascular bundle during laser irradiation; besides, contact with the maxillary bone should be avoided when using CO2 lasers on account of the risk of thermal damage. In addition, it must be taken into account that muscle fibers are thicker in the vestibular zone, while it is advisable not to go beyond the limits of the frenulum insertion area during irradiation.

The operating power of the CO2 laser was set at 5 W, since sectioning and vaporization at lower settings were very slow, but too fast at higher settings.

Patients requiring upper lip lengthening or bone surgery were excluded, despite the fact that the Er,Cr:YSGG laser can also be used in ostectomy procedures.

Considering the results obtained in our study, it should be underscored that postoperative periods were painless (except in two patients) and with no swelling, regardless of the type of laser used. Different authors agree that the postoperative course is very comfortable for patients, since it is the result of a reduced swelling and a minimal inflammatory response. This can be explained by the scant laser damage caused to adjacent tissues, the sealing of lymphatics, and the formation of a fibrin clot over the surgical wound that protects the wound from external irritation (17-19).

Many authors (20,21) have also reported the absence of immediate postoperative pain after oral mucosal surgery with lasers, so it has been suggested that this pain-free scenario can be attributed to the fact that laser irradiation seals nerve endings, which are therefore unable to develop anastomosis among each other.

The efficiency of the CO2 laser allows sealing lymphatic and blood vessels using a caliber beam under 0.5 mm that renders a bloodless surgical field. This may account for the minimal fluid extravasation observed, with a bloodless surgery and a minimal inflammatory response around the tissues under surgery management.

In our series the Er,Cr:YSGG laser achieved faster healing than the CO2 laser (2 weeks and 3 weeks, respectively). However, no studies in the literature have compared differences in healing times between these two laser systems.

In conclusion, the use of either type of laser is useful for a simple frenectomy due to their numerous advantages. The most important benefits are as follows: technical simplicity, with a short operating time; absence of postoperative swelling and pain; and the achievement of a correct upper lip frenulum reinsertion at the MGJ in all the patients included in our series at 4 months after the frenectomy procedure.
